# Dynamic Behavior Analysis of Complex-Configuration Organic Rankine Cycle Systems Using a Multi-Time-Scale Dynamic Modeling Framework

**DOI:** 10.3390/e27111170

**Published:** 2025-11-19

**Authors:** Jinao Shen, Youyi Li

**Affiliations:** School of Marine Engineering Equipment, Zhejiang Ocean University, Zhoushan 316022, China; shenjinao0205@zjou.edu.cn

**Keywords:** multi-time-scale, organic Rankine cycle, dynamic model, complex configuration

## Abstract

Organic Rankine Cycle (ORC) systems with complex configurations exhibit strong thermo-mechanical–electrical–magnetic coupling, making dynamic analysis computationally demanding. This study proposes a multi-time-scale modeling framework that partitions the system into second-, decisecond-, and hybrid-scale subsystems for separate computation, reducing simulation time while maintaining accuracy. Dynamic models are developed for heat exchangers, expanders, pumps, generators, and converters. The method is validated on a basic ORC system using operational data, achieving a mean absolute error of 2.12%, well within the ±5% tolerance. It is then applied to a series dual-loop ORC and a multi-heat-source ORC with series heat exchangers. Results indicate that the dual-loop configuration enhances disturbance rejection to both sink and heat-source fluctuations, while dual-heat-source system dynamics are predominantly governed by the second heat source. The framework enables efficient, accurate simulation of complex ORC architectures and provides a robust basis for advanced control strategy development.

## 1. Introduction

Global warming poses a severe threat to human life, prompting nations worldwide to set targets for achieving net-zero carbon emissions [1]. Over 72 % of the thermal energy from fossil fuel combustion is released into the environment [2], while abundant renewable energy sources exist as low-grade heat, such as ocean thermal, geothermal, and solar thermal energy. Recovering low-grade heat is therefore an important strategy for reducing emissions and conserving energy [3]. The Organic Rankine Cycle (ORC) is a proven technology for converting low-grade heat into useful power [4]. However, the dynamic behavior of ORC systems is inherently complex due to nonlinear phase-change processes and strong parameter coupling, making both dynamic analysis and control system design challenging [5].

Therefore, dynamic modeling plays a critical role in ORC control design. Quoilin et al. [6] developed a dynamic ORC model and evaluated three control strategies, showing that model predictive control (MPC) performed best under variable operating conditions. Desideri et al. [7] used the ThermoCycle library in Dymola to build a dynamic model with minimal steady-state and transient errors. Xu et al. [8] coupled a diesel engine and an ORC system to simulate transient responses. Chen et al. [9] modeled an ORC with mixed working fluids and proposed three control strategies. Cai et al. [10] examined the impact of varying fluid compositions on system dynamics. Zhou et al. [11] applied a validated transient model to assess five strategies under thermal efficiency and operational constraints. Gu et al. [12] optimized ORC performance using a dynamic model.

Consequently, further studies have explored diverse modeling approaches. Wei et al. [13] employed Modelica to simulate ORC performance under steady-state and small disturbances. Casella et al. [14] modeled a 150 kW ORC in Modelica with strong agreement in both steady-state and dynamic regimes. Bamgbopa and Uzgoren [15] proposed a quasi-dynamic model with a dynamically modeled evaporator and steady-state pump/expander, validated under variable heat-source conditions. Huster et al. [16] constructed a comprehensive ORC model with moving-boundary heat exchanger models and steady-state components, achieving accurate validation results. Wang et al. [17] integrated a dynamic turbine model, reducing simulation error relative to static models. Shi et al. [18] applied the Koopman operator to reduce computational load while maintaining accuracy. Zhang et al. [19] analyzed combined heat-and-power ORC dynamics, and Dhanasegaran et al. [20] used a thermal-resistance network for transient characterization.

The literature indicates that most ORC dynamic modeling focuses on simple configurations [5], with few studies addressing complex layouts. ORC systems with complex configurations exhibit superior performance [21]; for example, dual-loop ORC systems offer superior performance compared with single-loop designs [22], and series heat exchanger configurations can harness multiple heat sources [23]. Sanaye and Ghaffari [24] conducted transient simulations on the dual-loop ORC system, in which the heat exchanger was modeled using the Finite Volume Method (FVM). The simulation results indicated that the single-loop ORC system has a shorter time to reach a steady state than the dual-loop counterpart. Lu et al. [25] developed a dynamic model of an ORC system, which can recover exhaust gas and coolants from vehicular engine. The heat exchanger model also utilized the Finite Volume Method (FVM). The simulation results demonstrated that mutual interference exists between the heat sources, with the influence of exhaust gas being more significant. However, complex ORC systems involve more heat exchangers and components, leading to intricate dynamic interactions and significantly higher computational costs for simulation. ORC systems with complex configurations exhibit strong thermo-mechanical–electrical–magnetic coupling, which greatly increases the computational burden of dynamic simulation. In particular, complex ORC architectures incorporating multiple heat exchangers and components introduce intricate thermal and mechanical interactions that render single-time-scale dynamic modeling computationally intensive and often impractical for system-level analysis.

To alleviate this problem, Gonidaki et al. [26] simulated a solar-driven ORC system by adopting steady-state models for heat exchangers while dynamically modeling only the thermal storage tank, thereby reducing simulation time. Similarly, Zheng et al. [27] applied steady-state models to all components in a dual-loop ORC system to minimize computational cost. These studies demonstrate that steady-state modeling is a widely used strategy to improve simulation efficiency. However, such simplifications inevitably compromise the ability to accurately reproduce transient behaviors, especially under rapid thermal or load fluctuations.

Therefore, a fundamental gap remains between computational efficiency and dynamic fidelity in the modeling of complex ORC systems. To bridge this gap, the present study proposes a multi-time-scale modeling framework that partitions the ORC system into subsystems characterized by distinct response times—specifically, second-, decisecond-, and hybrid-scale components—for separate computation. This approach enables high-accuracy dynamic simulation while significantly reducing computational cost. The framework is validated using operational data from a basic ORC system, and then extended to dual-loop and multi-heat-source ORC configurations with series-connected heat exchangers. The results demonstrate that the dual-loop configuration improves disturbance rejection to both heat-source and sink variations, while the multi-heat-source system dynamics are primarily governed by the secondary heat source. Overall, the proposed framework provides an efficient and accurate tool for analyzing the dynamic behavior of complex ORC systems and lays a solid foundation for the development of advanced control strategies.

## 2. Dynamic Modeling

A computationally efficient and numerically accurate dynamic model of an ORC system facilitates the investigation of system dynamics and enables robust controller design for complex configurations. However, conventional single-time-scale modeling approaches suffer from long computational times because they simultaneously resolve all system components, regardless of their distinct dynamic response times. This study proposes a multi-time-scale modeling methodology for ORC systems, in which the simulation is partitioned according to the dynamic response times of individual components.

### 2.1. Description of the ORC

#### 2.1.1. Basic ORC

**Figure 1:**
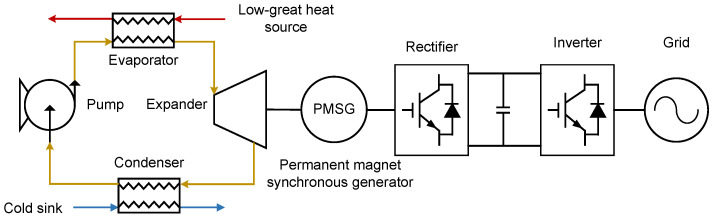
Layout of basic ORC system.

**Figure 2:**
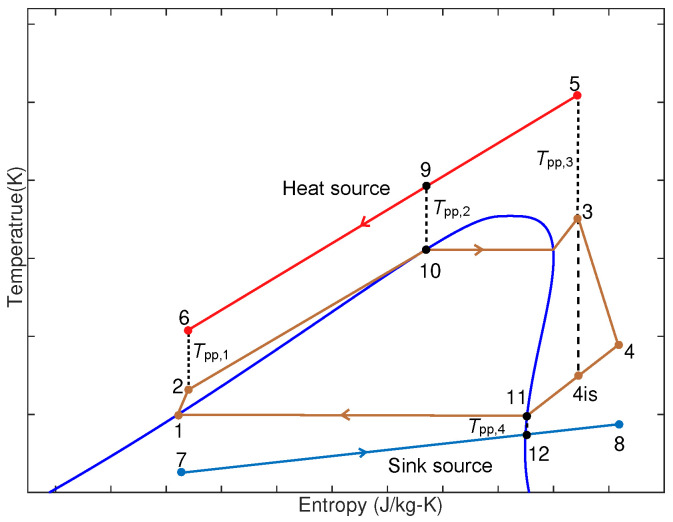
T-s diagram of basic ORC system.

The Organic Rankine Cycle (ORC) is regarded as one of the most promising technologies for low-grade thermal energy power generation [28]. Its configuration is analogous to that of the conventional steam Rankine cycle, except that the working fluid is replaced by an organic refrigerant. The general architecture of an ORC system is shown in Figure 1. As illustrated, the system comprises an evaporator, pump, expander/turbine, condenser, generator, rectifier, and inverter. The corresponding temperature–entropy (T–s) diagram of a basic ORC is presented in Figure 2. This diagram depicts the thermodynamic processes in terms of temperature–entropy relationships, enabling evaluation of cycle efficiency and characterization of phase-change behavior in ORC systems.

#### 2.1.2. Multi-Loop ORC

**Figure 3:**
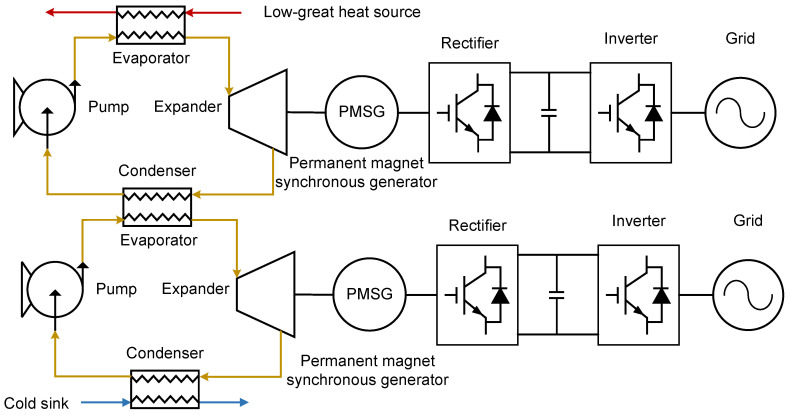
Layout of Series-Loop ORC system.

The multi-loop single-heat-source Organic Rankine Cycle system employs a series-loop configuration to maximize the extraction of thermal energy from a single heat source [29]. This configuration enhances thermodynamic performance—as evidenced by increased net power output and reduced exergy destruction—while lowering the capital expenditure per unit of generated power compared with single-loop systems. A schematic diagram of the series-connected multi-loop ORC system is shown in Figure 3.

Each loop in the series-loop ORC system is a fundamental ORC. In the primary loop, the following occur:

- The working fluid is pumped as a subcooled liquid into the evaporator, absorbing heat from the low-grade heat source, and become high-pressure superheated vapor.
- The vapor expands through the expander, converting thermal energy into mechanical work, and change into low-pressure superheated vapor.
- The resulting low-pressure vapor enters the intermediate heat exchanger, where it transfers residual heat to the secondary loop. Working fluid in the primary loop is condensed into a subcooled liquid, whereas the working fluid in the secondary loop is heated into superheated gas.
- The subcooled liquid is then recirculated to the evaporator via working fluid pump.

Critically, the condenser of the primary loop serves as the evaporator for the secondary loop, with heat exchange between loops occurring exclusively through the intermediate heat exchanger. This thermal cascading reduces exergy destruction by closely matching the temperature profiles of the working fluids.

#### 2.1.3. Multi-Source ORC

**Figure 4:**
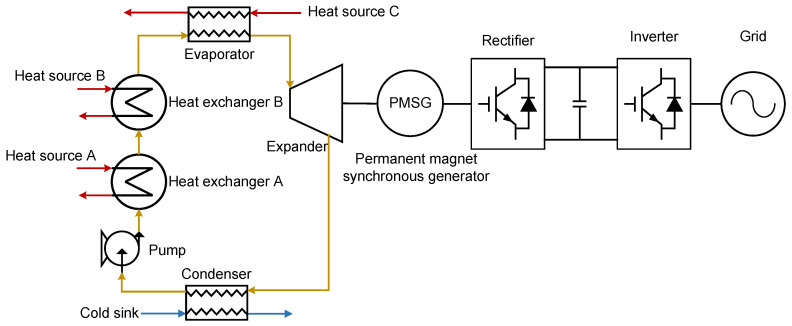
Layout of multi-heat-source ORC system.

A multi-heat-source ORC system with a series-connected heat exchanger configuration recovers thermal energy from multiple distinct sources within a single cycle, boosting net power output and reducing capital expenditure per unit of generated power through thermal integration [30]. As shown in Figure 4, the working fluid passes sequentially through multiple heat exchangers, absorbing heat from each source in turn.

As shown in Figure 4, subcooled liquid from the pump enters the first heat exchanger, where it is heated by heat source A. The preheated fluid then flows into the second heat exchanger, receiving additional thermal energy from heat source B. In the third heat exchanger, the fluid completes evaporation and superheating, becoming high-pressure superheated vapor. This vapor expands through the expander, producing mechanical work while transitioning to low-pressure superheated vapor. Finally, it is condensed in the condenser, rejecting heat to the cooling sink and returning to a subcooled liquid state.

Compared with conventional single-loop ORC systems, the series-connected heat exchanger configuration increases only the number of heat exchangers, while fully reusing existing components such as the pump, turbine, and condenser. This approach minimizes capital costs through hardware consolidation. Moreover, thermodynamic optimization—via heat-source matching, working fluid selection, and operational parameter tuning—significantly improves exergetic efficiency by reducing thermal irreversibilities across the temperature gradient.

### 2.2. Evaporator and Condenser

**Figure 5:**
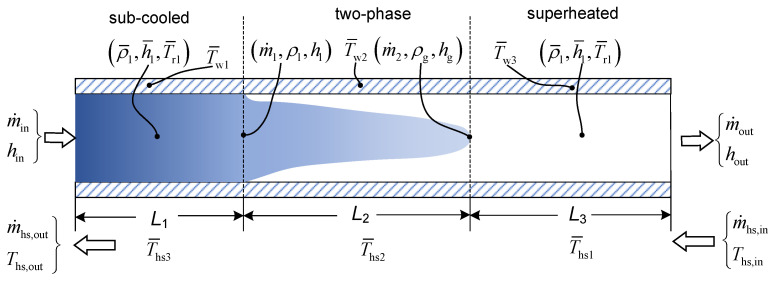
Moving boundary model for evaporator and condenser.

The evaporator and condenser are critical components responsible for exchanging energy with waste heat, and their characteristics strongly influence the overall dynamic performance of the ORC system. The phase-change processes occurring within these components are highly complex, exhibiting strong coupling and nonlinear behavior. To satisfy controller design requirements, this study adopts a moving boundary model (MBM) for the dynamic simulation of both the evaporator and the condenser [31]. In the MBM framework, as shown in Figure 5, each heat exchanger is divided into three distinct regions—subcooled liquid, two-phase, and superheated—separated by virtual boundaries that shift dynamically with changing operating conditions, and can be. A lumped-parameter approach is applied to model each region individually. To balance computational efficiency with model fidelity, the following assumptions are made:

- The pressure drop of the working fluid within the heat exchanger is assumed to be negligible;
- The working fluid flow within the heat exchanger tubes is modeled as an idealized one-dimensional flow;
- The axial conduction of both the working fluid and plate walls along the flow direction is neglected;
- The influence of gravitational forces on the working fluid flow is disregarded in the present model.

Under the aforementioned assumptions, and neglecting the momentum conservation equation, the nonlinear differential equation governing the working fluid flow can be expressed as

(1) $$\begin{array}{cc} \frac{\partial A\rho }{\partial t}+\frac{\partial \dot{m}}{\partial z} & =0 \end{array}$$

(2) $$\begin{array}{cc} \frac{\partial (\rho Ah-AP)}{\partial t}+\frac{\partial \dot{m}h}{\partial z} & =\pi D_{i}\alpha_{i}\left(T_{w}-T_{r}\right) \end{array}$$

where *A* is heat transfer area, ρ is density, $\dot{m}$ is mass flow rate, *h* is enthalpy of the working fluid, *P* is pressure, *D* is diameter, α is heat transfer coefficient, *T* is temperature, subscript w is the wall of heat exchanger, and subscript r is working fluid.

By integrating the mass and energy conservation equations across the three regions along the horizontal flow path, the mass and energy conservation equation of working fluid and wall for the each region can be obtained. The equations can be found in Appendix A.1. The heat transfer coefficients of working fluid and heat source in each zone are presented in Appendix A.2.

Geometric constraint of the heat exchanger:

(3) $$L=L_{1}+L_{2}+L_{3}$$

Consequently, the dynamic characteristics of the heat exchanger can be described by the following state variables:

(4) $$x=\left[L_{1},L_{2},h_{\mathrm{out}},p,{\bar{T}}_{w2},{\bar{T}}_{w2},{\bar{T}}_{w3}\right]$$

### 2.3. Expander

The scroll expander is a critical subsystem in ORC systems, converting the enthalpy of superheated vapor into mechanical work via isentropic expansion. In the integrated system architecture, the mass flow rate at the evaporator outlet is dynamically determined by the expander’s operational characteristics. Accordingly, the suction flow rate of the expander can be expressed as [6]

(5) $$m_{\mathrm{in},\exp}=\frac{F\;F\cdot N_{\exp}\cdot V_{\exp}}{60\cdot v_{r,\exp}}$$

where FF is the filling factor, $N_{\exp}$ is rotational speed, $V_{\exp}$ is displacement volume, and $v_{\exp}$ is specific volume of the working fluid at the expander inlet.

The expander model can be divided into two successive processes: isentropic expansion and constant volume expansion. The work performed in the isentropic process is expressed as

(6) $$W_{\exp,1}={\dot{m}}_{r}\left(h_{\exp,\mathrm{in}}-h_{\mathrm{is},\mathrm{out}}\right)$$

where $h_{\exp,\mathrm{in}}$ is the specific enthalpy of the working fluid at the expander inlet, and $h_{\mathrm{is},\mathrm{out}}$ is the isentropic enthalpy after the isentropic process.

The work performed in the constant volume expansion process is derived by

(7) $$W_{\exp,2}={\dot{m}}_{r}\cdot v_{r,\exp}\left(P_{\exp,\mathrm{in}}-P_{\exp,\mathrm{out}}\right)$$

where $v_{r,\exp}$ is the specific volume of the working fluid at the expander inlet, $P_{\exp,\mathrm{in}}$ is the pressure at inlet of the expander, and $P_{\exp,\mathrm{out}}$ is the pressure at the outlet of the expander. For $W_{\exp,2}$, it is positive with over-expansion and negative with under-expansion.

Summing up the work performed in the isentropic expansion and constant volume expansion yields the total expansion work of the expander. Considering other power losses of the expander, these losses are accounted for by introducing a mechanical efficiency $\eta_{\exp}$. Then, the output power of the expander is calculated as follows:

(8) $$\begin{array}{cc} W_{\exp} & =\left(W_{\exp,1}-W_{\exp,2}\right)\eta_{\exp}\\ \; & ={\dot{m}}_{r}\left(h_{\exp,\mathrm{in}}-h_{\mathrm{is},\mathrm{out}}+v_{r,\exp}\left(P_{\exp,\mathrm{in}}-P_{\exp,\mathrm{out}}\right)\right)\eta_{\exp} \end{array}$$

The shaft torque output is computed by

(9) $$T_{m}=\frac{W_{\exp}}{\omega_{\exp}}$$

where $\omega_{r}$ is shaft angular velocity.

### 2.4. Pump

The working fluid pump is a critical circulation component in ORC systems, delivering the closed-loop working fluid to the evaporator. The mass flow rate of the working fluid pump can be calculated using the following expression [19]:

(10) $${\dot{m}}_{\mathrm{pu}}=\eta_{\mathrm{pu}}\cdot \rho_{r,l}\cdot V_{\mathrm{pu}}\cdot \omega_{\mathrm{pu}}$$

where subscript pu is the pump.

The process undergone by the working fluid in the pump is an isentropic compression process; therefore, the enthalpy of working fluid at the pump outlet is calculated by the following formula:

(11) $$h_{\mathrm{pu},\mathrm{out}}=h_{\mathrm{pu},\mathrm{in}}+\frac{h_{\mathrm{is},\mathrm{pu},\mathrm{out}}-h_{\mathrm{pu},\mathrm{in}}}{\eta_{\mathrm{pu},\mathrm{is}}}$$

where the isentropic efficiency of the pump $\eta_{\mathrm{pu},\mathrm{is}}$ is obtained by fitting actual empirical data.

### 2.5. Permanent Magnet Synchronous Generator

Permanent magnet synchronous generators (PMSGs) offer high power density and strong reliability, making them the preferred choice for generators in ORC systems. For both steady-state and transient analyses of PMSGs, mathematical models in the dq coordinate system are generally employed. In this model, the *q*-axis is defined to lead the *d*-axis by $90^{\circ }$ electrical degrees, with the *d*-axis aligned along the rotor flux linkage direction. Accordingly, the voltage equations of the PMSG can be expressed as [32]

(12) $$\left\{\begin{array}{cc} u_{d} & =R_{s}i_{d}+L_{d}\frac{di_{d}}{dt}-\omega_{e}\psi_{q}\\ u_{q} & =R_{s}i_{q}+L_{q}\frac{di_{q}}{dt}+\omega_{e}\psi_{d} \end{array}\right.$$

where *u* is voltage, *R* is resistance, *L* is inductance, *i* is current, ψ is flux linkage, and subscript $\omega_{e}$ is electrical angular velocity.

$\psi_{q}$, $\psi_{d}$ can be calculated by

(13) $$\left\{\begin{array}{cc} \psi_{d} & =\psi_{f}+L_{d}i_{d}\\ \psi_{q} & =L_{q}i_{q} \end{array}\right.$$

Substituting Equation (13) into Equation (12) yields the following consolidated voltage–current relationship:

(14) $$\left\{\begin{array}{cc} u_{d} & =R_{s}i_{d}+L_{d}\frac{di_{d}}{dt}-\omega_{e}L_{q}i_{q}\\ u_{q} & =R_{s}i_{q}+L_{q}\frac{di_{q}}{dt}+\omega_{e}\psi_{f}+\omega_{e}L_{d}i_{d} \end{array}\right.$$

The electromagnetic torque of the permanent magnet synchronous generator (PMSG) is derived from the co-energy principle as follows:

(15) $$T_{e}=\frac{3}{2}p_{n}\left(\psi_{f}i_{q}+\left(L_{d}-L_{q}\right)i_{q}i_{d}\right)$$

where $p_{n}$ represents the pole-pairs.

Assuming that the PMSG is a surface-mounted type, the torque equation can be simplified as

(16) $$T_{e}=\frac{3}{2}p_{n}\psi_{f}i_{q}$$

The rotational dynamics of the PMSG is governed by

(17) $$J\frac{d\omega_{r}}{dt}=T_{e}-T_{m}$$

where *J* is the moment of inertia.

### 2.6. Converter

**Figure 6:**
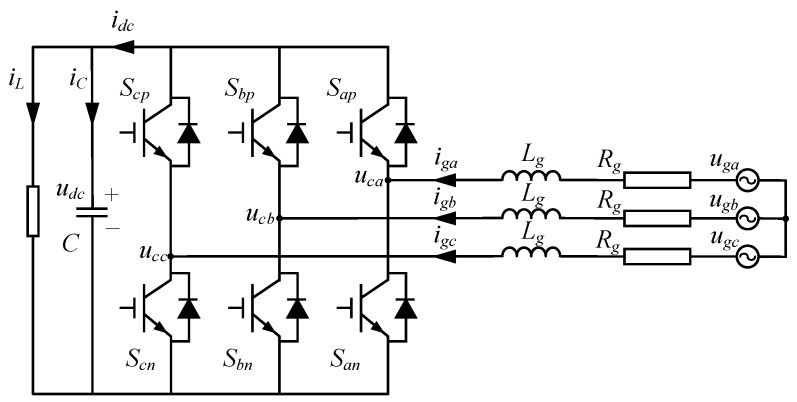
Configuration of PWM converter.

The converter is a key component in ORC power generation systems. The machine-side converter (MSC) regulates the rotational speed of the expander while simultaneously enabling active–reactive power decoupling. The grid-side converter (GSC) provides grid synchronization and regulates the DC-link voltage and power quality. Owing to the structural and control-theoretic similarities between the MSC and GSC—both employing dq-frame transformations and identical PWM switching principles—this section presents the GSC mathematical model as the representative case. The configuration of the PWM converter is shown in Figure 6.

According to Kirchhoff’s voltage law, the voltage relationship in the abc coordinate system can be obtained as follows [33]:

(18) $$\left\{\begin{array}{c} u_{ga}=L_{g}\frac{di_{ga}}{dt}+R_{g}i_{ga}+\left(S_{a}-\frac{S_{a}+S_{b}+S_{c}}{3}\right)u_{dc}\\ u_{gb}=L_{g}\frac{di_{gb}}{dt}+R_{g}i_{gb}+\left(S_{b}-\frac{S_{a}+S_{b}+S_{c}}{3}\right)u_{dc}\\ u_{gc}=L_{g}\frac{di_{gc}}{dt}+R_{g}i_{gc}+\left(S_{c}-\frac{S_{a}+S_{b}+S_{c}}{3}\right)u_{dc} \end{array}\right.$$

where *S* is the state of the switching device, 0 represents the off state, 1 represents the on state, and subscript *g* represents the grid.

DC-link capacitor dynamics:

(19) $$C\frac{du_{dc}}{dt}=S_{a}i_{ga}+S_{b}i_{gb}+S_{c}i_{gc}-i_{L}$$

Subsequently, the three-phase grid voltages and currents are transformed to the grid-synchronized dq reference:

(20) $$\left\{\begin{array}{c} u_{cd}=u_{gd}-R_{g}i_{gd}-L_{g}\frac{di_{gd}}{dt}+\omega L_{g}i_{gq}\\ u_{cq}=u_{gq}-R_{g}i_{gq}-L_{g}\frac{di_{gq}}{dt}-\omega L_{g}i_{gd} \end{array}\right.$$

(21) $$C\frac{du_{dc}}{dt}=S_{d}i_{gd}+S_{q}i_{gq}-i_{L}$$

### 2.7. Multi-Time-Scale Modeling Methodology

**Figure 7:**
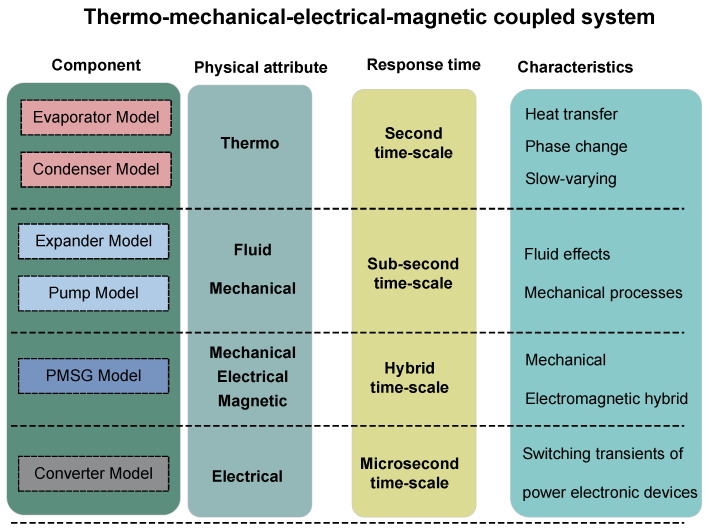
Multi-time-scale dynamic modeling framework.

Analysis of the mathematical models for each component in the aforementioned ORC power generation system indicates that it is a strongly coupled thermo-mechanical– electrical–magnetic system. The components exhibit markedly different response times when subjected to disturbances. Simultaneously solving the dynamic equations of all components inevitably leads to excessively long computation times. Therefore, based on the response times of individual devices and parameters, this study proposes a multi-time-scale modeling approach for the thermo-mechanical–electrical–magnetic coupled ORC power generation system, as illustrated in Figure 7.

As illustrated in Figure 7, the proposed methodology decomposes the ORC system into three distinct temporal scales to improve computational efficiency:

1. **Second-scale dynamics**
  This time-scale layer is the slowest and governs coupled heat transfer and phase-change processes in the evaporator and condenser subsystems. Characterized by slow-varying thermal behavior driven by thermal inertia, this scale exhibits time constants exceeding 1s.
2. **Decisecond-scale dynamics**
  This intermediate scale encompasses fluid–mechanical processes, including working fluid flow and turbomachinery rotation, and resolves the expander and working fluid pump models. Its dynamics are approximately one order of magnitude faster than those of the thermal subsystem owing to reduced inertial effects.
3. **Hybrid-scale electromagnetic transients**
  The PMSG operates over a hybrid time-scale. For front-end dynamics (fluid–mechanical coupling with the ORC unit), it is temporally coupled with the decisecond-scale subsystem. Conversely, for back-end dynamics (grid interaction via the power converter), it must be resolved concurrently with the electromagnetic transients of the converter. This dual-scale treatment captures the PMSG’s bidirectional coupling across the mechanical and electrical domains.

### 2.8. Model Development

#### 2.8.1. Parameter Interaction

This study develops and numerically solves the dynamic model using the proposed multi-time-scale modeling framework. The parameter interactions between components in the dynamic model of the basic ORC system are illustrated in Figure 7.

**Figure 8:**
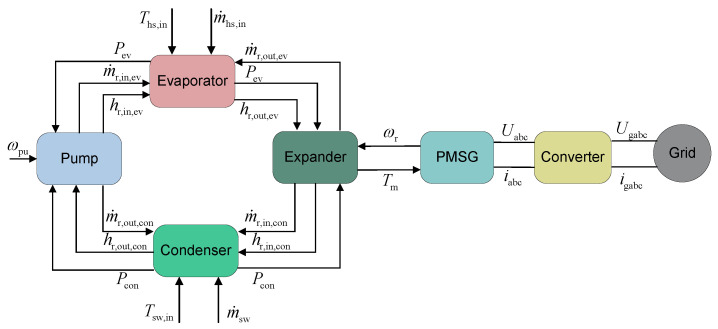
Parameter interaction of basic ORC system.

Figure 8 presents the parameter interactions among various components of the ORC system during the solution process. In the evaporator model, the mass flow rate and enthalpy of the working fluid at the inlet are provided by the working fluid pump, while the mass flow rate of the working fluid at the outlet is determined by the turbine. The evaporator model supplies the outlet pressure to the working fluid pump as well as the inlet pressure and enthalpy of the working fluid to the turbine. For the turbine, the inlet pressure and enthalpy of the working fluid are provided by the evaporator, and the turbine supplies the outlet mass flow rate to the evaporator. Meanwhile, the turbine delivers the mass flow rate and enthalpy of the working fluid to the condenser, and the outlet pressure of the turbine is provided by the condenser. Similar to the evaporator model, the condenser model mainly provides the inlet pressure to the working fluid pump and the outlet pressure to the turbine. The mass flow rate and enthalpy of the working fluid at the inlet of the working fluid pump are provided by the condenser, and the working fluid pump supplies the inlet mass flow rate and enthalpy of the working fluid to the evaporator.

Furthermore, the rotational speed of the turbine is determined by the PMSG, while the turbine provides its rotational speed to the PMSG. The PMSG supplies voltage to the converter, and the converter provides current to the PMSG. Additionally, the converter and the power grid interact with each other regarding voltage and current parameters.

**Figure 9:**
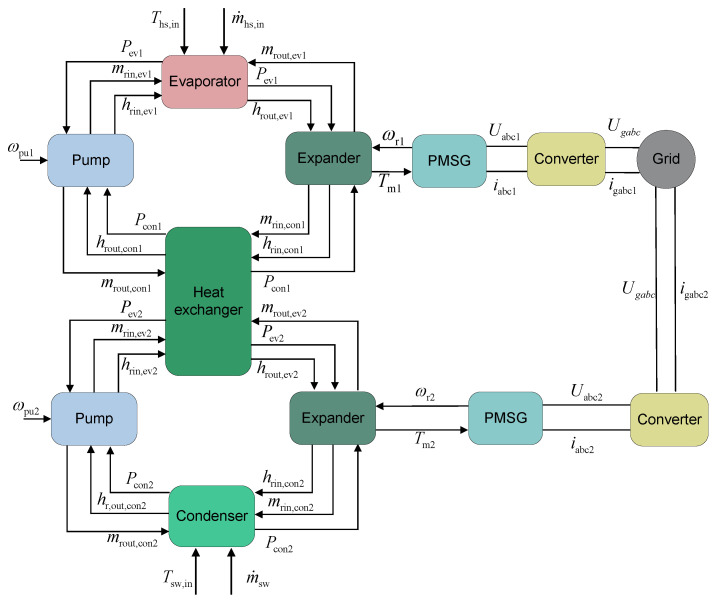
Parameter interaction of series-connected dual-loop ORC system.

In the dynamic model of a series-loop ORC system, the parameters governing component coupling are illustrated in Figure 9. The intermediate heat exchanger acts as the condenser for the primary loop while serving as the evaporator for the secondary loop.

Figure 9 illustrates the parameter interaction among various components in the series-connected dual-loop ORC system during the solution process. This system is highly similar to the basic ORC system, with the key difference lying in the intermediate heat exchanger. Specifically, the intermediate heat exchanger serves as the condenser for the first loop while functioning as the evaporator for the second loop. Within the intermediate heat exchanger, it provides the pressure and enthalpy of the working fluid at the inlet to the working fluid pump of the first loop, and supplies the outlet pressure to the working fluid pump of the second loop. Furthermore, for the intermediate heat exchanger model, it delivers the outlet pressure of the working fluid to the turbine in the first loop, and provides the inlet pressure to the turbine in the second loop.

**Figure 10:**
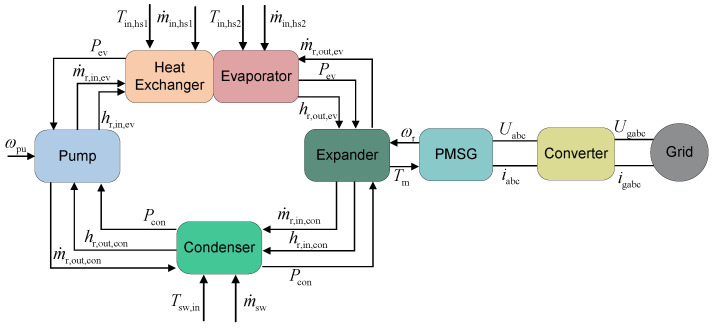
Parameter interaction of multi-heat-source ORC system.

In the dynamic model of a dual-heat-source ORC system, the intra-component parameter coupling is shown in Figure 10.

As can be observed in Figure 10, the parameter interaction among various components of the dual-heat-source ORC system is illustrated. The key difference from the basic ORC system lies in the fact that the evaporator is replaced by a combination of a heat exchanger and an evaporator. Specifically, it is necessary to clarify the parameter interaction between the heat exchanger and the evaporator. During the solution process, for the heat exchanger, its pressure is provided by the evaporator, while it supplies the inlet mass flow rate and enthalpy of the working fluid to the evaporator.

#### 2.8.2. Time-Scale Selection

In the dynamic model of the heat exchanger, the pressure variable exhibits relatively rapid fluctuations. However, its response is constrained by the heat transfer and phase-change processes. As a result, the simulated rate of pressure variation is comparatively slow, and the corresponding time-scale is on the order of seconds. Similarly, enthalpy is influenced by both heat transfer and pressure variations, and thus its simulation is also conducted on the second-order time-scale.

For the expander model, the fluid and mechanical responses are faster than the heat transfer processes in the heat exchanger. Nevertheless, the mechanical and fluid parameters within the expander are affected by the operating conditions of the evaporator and condenser. Therefore, in analyzing the dynamic characteristics of the ORC system, the expander and heat exchanger models are solved simultaneously. During simulation, a second-order time-scale is adopted. If the time step is excessively large, abrupt parameter variations may occur during the solution process, leading to divergence. Conversely, if the time step is too small, computational costs increase substantially, and the cumulative error in the heat exchanger mass flow rate may eventually result in mass imbalance.

When investigating the characteristics of electrical components, the response time of electrical parameters is significantly faster than that of mechanical ones. Accordingly, the expander, PMSG, and converter are solved using two separate time-scales during the simulation. Specifically, the expander model is computed on a sub-second time-scale, while the converter and PMSG are resolved on a microsecond scale. If the entire system were solved on the microsecond scale, computational resources would be wasted unnecessarily. On the other hand, if the electrical device models were also solved on the sub-second scale, their fast dynamics could not be captured accurately, resulting in substantial errors.

When performing overall simulations of an ORC system integrated with electrical devices, the solution process must be partitioned into three distinct time-scales. The heat exchangers are modeled on a second-order time-scale, the pumps and expander on a sub-second scale, and the PMSG together with the converter on a microsecond scale. Applying a single uniform time-scale to the entire system would result in substantial inefficiencies and severe waste of computational resources.

The interaction among components in the ORC system occurs across multiple time-scales, leading to coupled dynamic behaviors that are essential to understand. The slow thermal response of the condenser influences the outlet pressure of the expander, thereby affecting its instantaneous power output and efficiency. Meanwhile, the evaporator dynamics determine the rate of pressure buildup and directly interact with the pump flow and expander torque. When rapid load fluctuations occur, mismatched time constants among the pump, expander, and heat exchangers can cause transient oscillations in evaporating pressure, superheat degree, and output power. Therefore, the multi-time-scale modeling framework not only distinguishes the temporal evolution of each component but also captures these physical dependencies, which are crucial for analyzing and controlling the dynamic behavior of complex ORC systems.

## 3. Results and Discussion

### 3.1. Parameters of ORC System

The heat sources are from experimental characterization of a marine low-speed two-stroke diesel engine operating at 75% load, as detailed in Table 1.

The heat exchanger parameters for the cascaded dual-loop ORC power generation system are listed in Table 2.

As can be seen in Table 2, a plate heat exchanger is selected as the intermediate heat exchanger in the series dual-loop ORC system. The reason is that the fluid in both sides of the heat exchanger are in liquid state, and plate heat exchangers possess a higher heat transfer coefficient.

Likewise, the heat exchanger parameters for the multi-heat-source ORC system are provided in Table 3.

The parameters of other components in the ORC system are presented in Table 4.

### 3.2. Model Validation

#### 3.2.1. Simulink Model Configuration and Solver Settings

The multi-time-scale dynamic model of the basic ORC system is developed in MATLAB/Simulink 9.10, with each device model implemented using S-functions. Within each S-function, the simulation time step for the corresponding device can be independently configured. To realize the proposed multi-time-scale partitioning, three solver configurations were adopted:

- **Second-scale subsystem (thermal domain):** A variable-step solver (ode23t, trapezoidal rule) with maximum step size of 1s and relative tolerance of $10^{-3}$.
- **Decisecond-scale subsystem (mechanical domain):** A fixed-step solver (ode4, Runge Kutta method) with a step size of 0.1s.
- **Hybrid-scale subsystem (electrical and converter domain):** A fixed-step discrete solver (Tustin method) with a sample time of $1\times 10^{-6}\;s$ to ensure accurate tracking of converter current and generator electromagnetic torque.

#### 3.2.2. Validation Results

**Figure 11:**
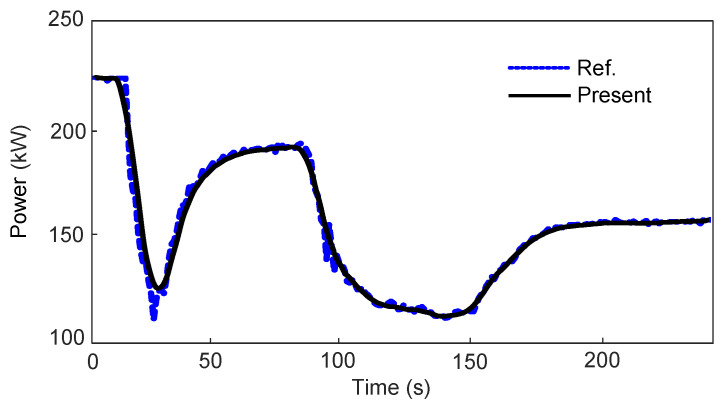
Validation of the overall dynamic model of the orc power generation system.

The overall ORC system model is validated against experimental data from Ref. [34], which characterizes the output power response under transient heat-source temperature variations. As shown in Figure 11, the simulated output power of the ORC power generation system closely matches the experimental data. The mean absolute error is 2.12%, and the peak error is 9.53%. This error profile supports the validity of the proposed temporal decoupling framework. The maximum deviation occurs when second-scale thermal dynamics temporarily dominate the decisecond-scale mechanical response. Under steady-state conditions and mild transients (temperature variation rates below 2 °C/s), the model maintains an accuracy better than 1.5%.

### 3.3. Dynamic Characteristics of the Series Dual-Loop ORC System

#### 3.3.1. Effect of Heat Source

**Figure 12:**
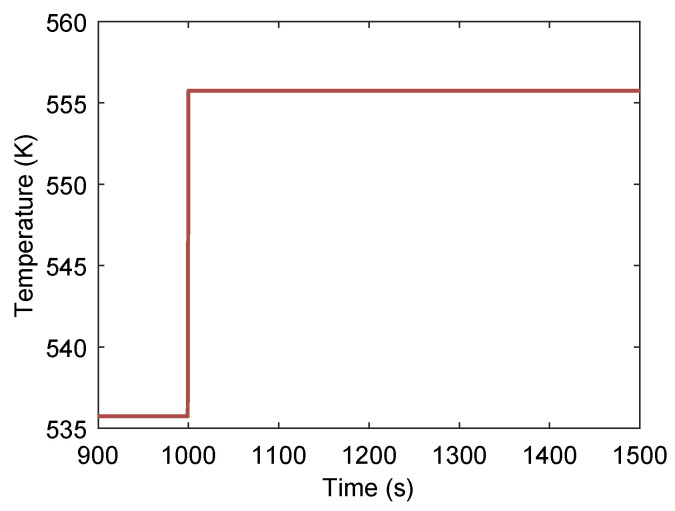
Heat-source temperature undergoes a step increase of 20 °C at t=1000s.

In the following section, the transient response of a series-configured dual-loop ORC power generation system to disturbances in both heat source and heat sink conditions is investigated. First, the variation in key system parameters is analyzed for a case in which the heat source temperature undergoes a step increase of 20 °C at t=1000s, as shown in Figure 12.

**Figure 13:**
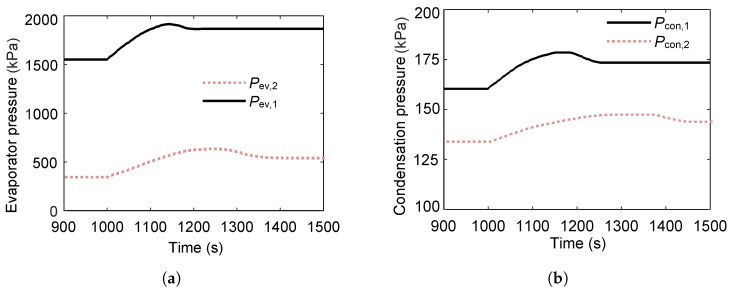
Pressure variations in the heat exchangers during a step increase in heat-source temperature. (**a**) Evaporating pressure response to the step change in heat-source temperature. (**b**) Condensing pressure response to the step change in heat-source temperature.

Figure 13 illustrates the transient pressure response within the heat exchangers of the series-coupled dual-loop ORC system following a 20 °C step increase in heat-source temperature at t=1000s. The rise in heat-source temperature increases the evaporating pressures in both the first and second loops. Compared with the first loop, the evaporating pressure in the second loop exhibits smaller fluctuations, primarily due to the thermal buffering effect of the intermediate heat exchanger, which damps rapid pressure variations. The relatively long phase-change duration in the second loop introduces a noticeable delay in the propagation of the stepwise thermal disturbance from the first loop. Similarly, the condensing pressures of both loops show a slight increase, although the second loop’s condensing pressure remains almost constant over time, reflecting the stabilizing influence of the condenser’s large thermal inertia and constant cooling sink conditions.

**Figure 14:**
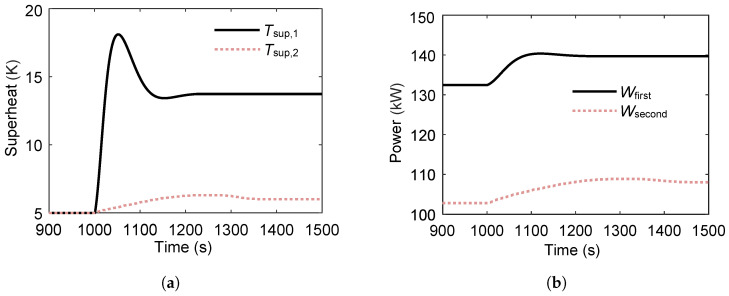
Superheat temperature and power output variations in the ORC system during a step increase in heat-source temperature. (**a**) Superheat temperature response to the step change in heat-source temperature. (**b**) Power output response to the step change in heat-source temperature.

As shown in Figure 14, the variations in superheat temperature and power output for the two circulation loops are examined during a stepwise increase in heat-source temperature. The superheat degree of the first loop exhibits pronounced fluctuations, whereas that of the second loop displays smaller fluctuations accompanied by a considerable delay. Specifically, the first loop’s superheat degree increases more substantially, while the magnitude of increase in the second loop is smaller. Figure 14b shows that the rise in heat-source temperature increases the power output of both cycles; however, the first loop experiences a more significant increase, whereas the second loop shows a more modest gain. This suggests that the additional heat transfer capacity from the elevated heat-source temperature is primarily absorbed by the first loop, making its performance more critical to the overall system output.

#### 3.3.2. Effect of Sink Source

Analysis of the dynamic characteristics of the series-type dual-loop ORC system under heat-source disturbances indicates that the first loop acts as a thermal buffer, mitigating the impact of heat-source fluctuations on the system. The next section examines variations in key parameters when the cold source temperature undergoes a stepwise decrease of 2 °C at t=1000s.

**Figure 15:**
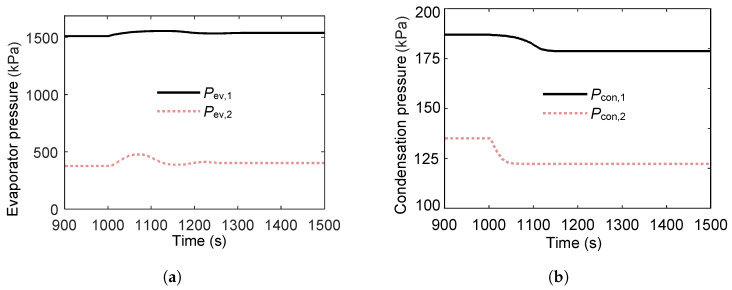
Pressure variations in the heat exchangers of the ORC system during a 2 °C step decrease in sink temperature at t=1000s. (**a**) Evaporating pressure response to the decrease in sink temperature. (**b**) Condensing pressure response to the decrease in cold source temperature.

As shown in Figure 15, a stepwise decrease in cold-source temperature causes both the evaporating and condensing pressures in the two cycles to decrease. The fluctuations in evaporating pressure are smaller than those in condensing pressure because cooling water temperature changes act directly on the condensers, producing a stronger and more immediate effect.

The evaporating pressure in the second loop exhibits a secondary fluctuation, caused by the first loop not reaching a quasi-steady state and transmitting an oscillatory thermal load to the second loop’s evaporator. This indicates that transient instability in the primary loop can propagate downstream, albeit with reduced magnitude.

The condensing pressure in the first loop shows smaller fluctuations, while the condensing pressure in the second loop stabilizes more quickly. This faster stabilization is attributed to the damping effect of the intermediate heat exchanger and the second loop’s lower sensitivity to upstream disturbances. Together, these characteristics enhance the second loop’s resilience to cold-source transients.

**Figure 16:**
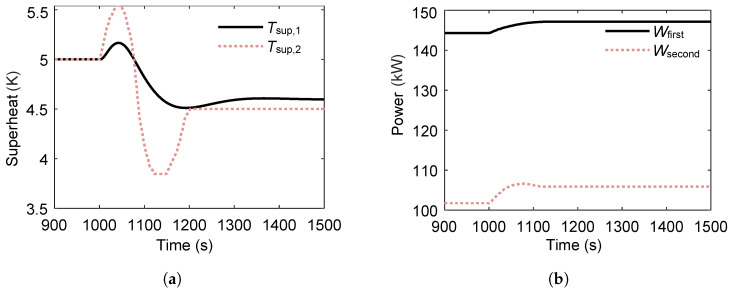
Transient response of superheat temperature and net power output in the ORC system during a 2 °C step decrease in sink temperature at t=1000s. (**a**) Superheat temperature response to the sink temperature decrease. (**b**) Net power output response to the sink temperature decrease.

Figure 16 shows the variations in superheat temperature and power output of the dual-loop ORC system following a 2 °C step decrease in sink temperature at t=1000s. The superheat temperature of the second loop exhibits more pronounced variations than that of the first loop due to its direct thermal coupling with the condenser affected by the sink temperature change. However, the second loop reaches a steady state faster, indicating a lower thermal inertia compared with the first loop. After steady state is reached, both loops experience a reduction in superheat temperature, with the decrease in the second loop being larger—reflecting its higher sensitivity to cold-source perturbations.

In terms of power output, both loops show an increase following the sink temperature decrease. The second loop’s power output increases by a larger proportion and at a faster rate than the first loop, owing to the greater enhancement in its condensation temperature difference and corresponding turbine expansion ratio.

From the combined analysis of the system’s dynamic characteristics under both heat- and cold-source disturbances, it is evident that the dual-loop ORC system exhibits stronger anti-disturbance capability than a single-loop configuration. Under heat-source fluctuations, the second loop experiences smaller performance variations due to the thermal buffering effect of the first loop. Under cold-source variations, the first loop is less affected, benefiting from the damping effect provided by the intermediate heat exchanger and the second loop’s thermal response characteristics.

#### 3.3.3. Effect of Expander Torque

**Figure 17:**
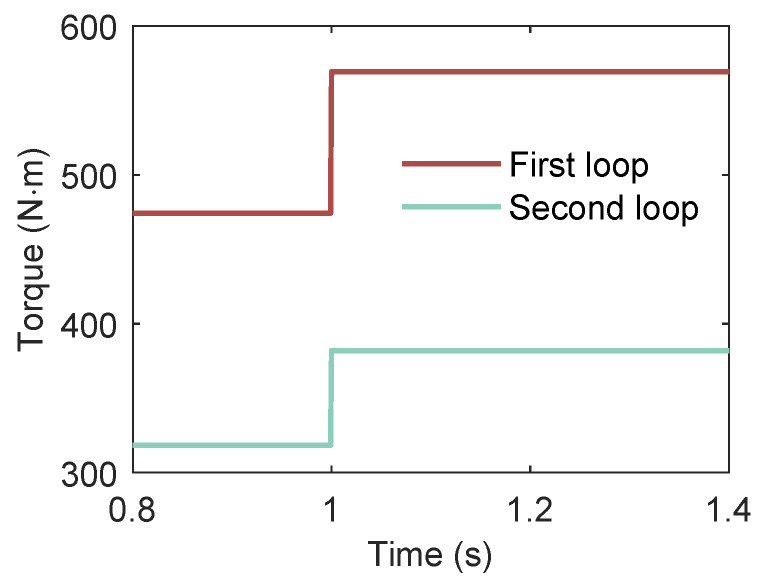
Expander mechanical torque of both loops undergo a 20% stepwise increase at t=1s.

**Figure 18:**
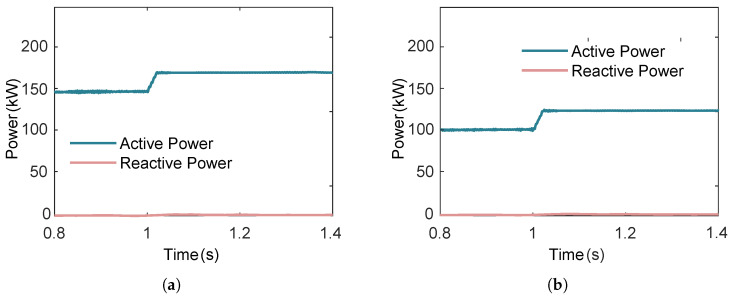
Transient response of the ORC system power output during a 20% step increase in expander mechanical torque at t=1s. (**a**) Power output of the first loop response to step increase in expander mechanical torque. (**b**) Power output of the second loop response to step increase in expander mechanical torque.

A series dual-loop ORC system contains two or more converters, where the power between the two loops is mutually coupled. Under grid-connected conditions, the multi-loop ORC system delivers the maximum power to the grid. Assuming that at 1 s, the expander mechanical torque of both loops undergo a 20% stepwise increase, as shown in Figure 17, the simulation results of the power output from the grid-side inverters of the two loops are as shown in Figure 18 below.

As can be observed in Figure 18, when the expander mechanical torque undergo a stepwise increase, the active power of both loops exhibits an upward trend. This is because the machine-side control strategy maintains the rotational speed at a given value, while the reactive power remains essentially unchanged.

### 3.4. Dynamic Characteristics of the Multi-Heat-Source ORC System

In the multi-heat-source ORC power generation system, two heat-sources—scavenge air (SA) and exhaust gas (EX)—supply thermal energy through three heat exchangers, with parameters listed in Table 1. Compared with the basic ORC system, the dual-heat-source configuration adds one heat exchanger without increasing or removing other components. Consequently, disturbance analysis for sink temperature is unnecessary.

#### 3.4.1. Effect of Multi-Heat-Source

When analyzing the dynamic characteristics of the dual-heat-source ORC system under heat-source disturbances, the differences between scenarios in which both heat-source temperatures change simultaneously (either increasing or decreasing) and those in which only one heat-source temperature changes are minimal. Therefore, the focus of this study is on cases where the two heat sources exhibit opposite variation trends. Two scenarios are considered:

Case 1: SA temperature increases by 20 °C at t=1000s while EX temperature decreases by 20 °C.
Case 2: SA temperature decreases by 20 °C at t=1000s while EX temperature increases by 20 °C.

**Figure 19:**
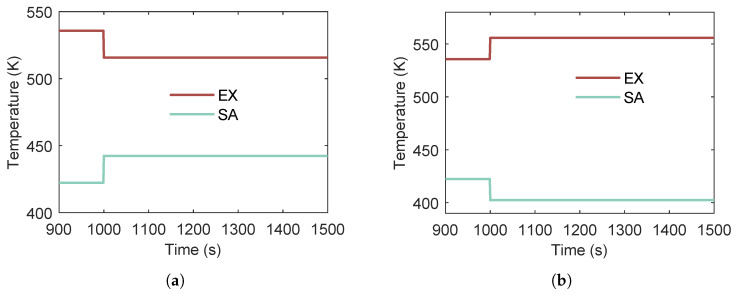
Two heat sources exhibit opposite variation trends. (**a**) SA temperature increases by 20 °C at t=1000s while EX temperature decreases by 20 °C. (**b**) SA temperature decreases by 20 °C at t=1000s while EX temperature increases by 20 °C.

The two cases can be seen in Figure 19.

**Figure 20:**
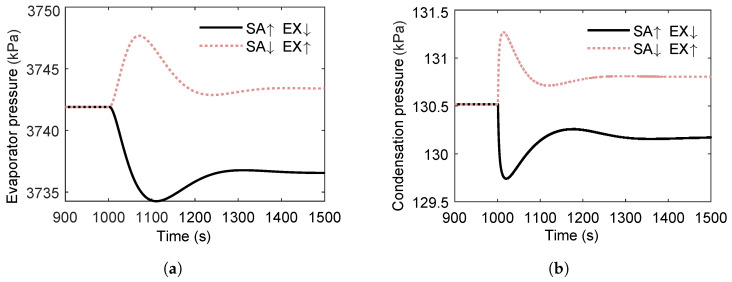
Pressure dynamics under anti-correlated dual-heat-source temperature perturbations in a dual-heat-source ORC system. (**a**) Evaporating pressure response to temperature variations of the two heat sources. (**b**) Condensing pressure response to temperature variations of the two heat sources.

Figure 20 shows the transient pressure responses within the heat exchangers when the two heat-source temperatures vary in opposite directions. The notation SA↑EX↓ denotes a simultaneous +20 °C step in scavenge air (SA) temperature and a −20 °C step in exhaust gas (EX) temperature, whereas SA↓EX↑ indicates the reverse pairing.

At the new steady state, the SA↓EX↑ case results in a modest increase in both evaporator and condenser pressures relative to the initial condition, while the SA↑EX↓ case yields a corresponding slight decrease. This behavior arises because the exhaust gas heat source is of inherently higher quality than the scavenge air heat source. Consequently, an increase in exhaust gas temperature raises the evaporating pressure above its initial value, even when accompanied by a simultaneous decrease in scavenge air temperature.

Moreover, both the evaporating and condensing pressures change more rapidly in the SA↓EX↑ case than in the SA↑EX↓ case. This difference is explained by the location of heat transfer: the exhaust gas supplies heat near the working fluid outlet, where the fluid is predominantly in the vapor phase, whereas the scavenge air heats the fluid in the liquid phase. Since the specific heat capacity of vapor is lower than that of liquid, its temperature rises more rapidly, leading to faster pressure dynamics.

**Figure 21:**
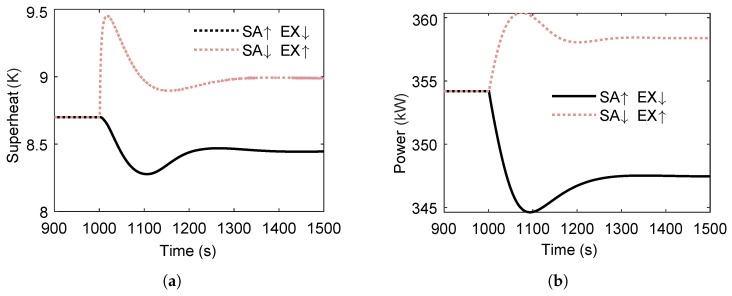
Variations in superheat temperature and power output under opposing temperature trends of dual heat sources. (**a**) Superheat temperature response to temperature variations of the two heat sources. (**b**) Power output response to temperature variations of the two heat sources.

Figure 21 shows the variations in superheat temperature at the heat exchanger outlet and power output when the two heat sources vary in opposite directions. Under the SA↓EX↑ condition, the superheat temperature rises rapidly, then decreases, and finally stabilizes at a value slightly higher than the initial state. In contrast, the SA↑EX↓ condition produces the opposite trend. This behavior can be explained by the different roles of the two heat sources in the cycle: scavenge air preheats the subcooled working fluid, where no phase change occurs and enthalpy increases slowly, whereas the exhaust gas dominates the evaporation and superheating processes. Consequently, changes in exhaust gas temperature have a direct impact on the outlet vapor state. However, due to thermal inertia, the rate of superheat temperature decrease in the SA↑EX↓ case is slower than the rate of increase in the SA↓EX↑ case.

In terms of power output, the SA↑EX↓ condition results in a pronounced decrease, while the SA↓EX↑ condition produces a modest increase. This asymmetry arises because the scavenge air is a lower-quality heat source, whereas high-temperature exhaust gas enables higher thermoelectric conversion efficiency.

#### 3.4.2. Effect of Expander Speed

**Figure 22:**
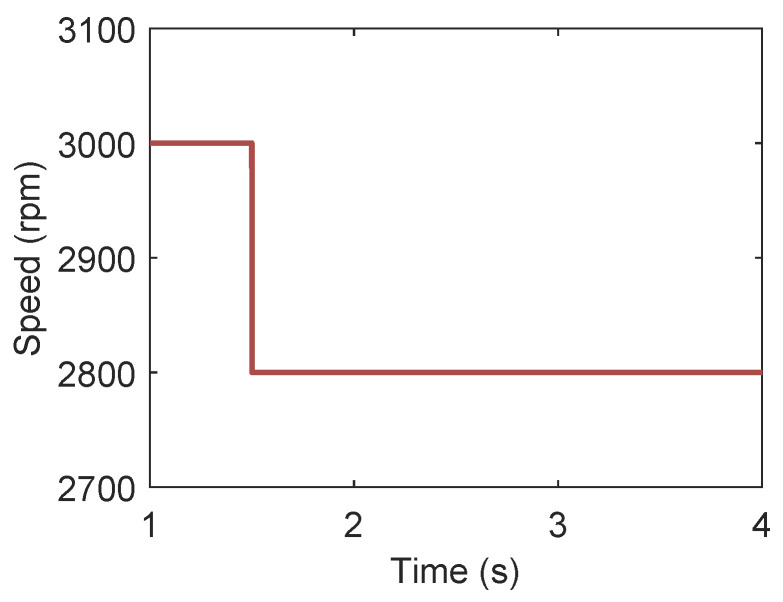
Expander speed changes from 3000 rpm to 2800 rpm at t=1s.

After the ORC system enters stable state, this section presents the variations in various parameters in the ORC system when the rotational speed changes from 3000 rpm to 2800 rpm at 1.5 s, which is illustrated in Figure 22.

**Figure 23:**
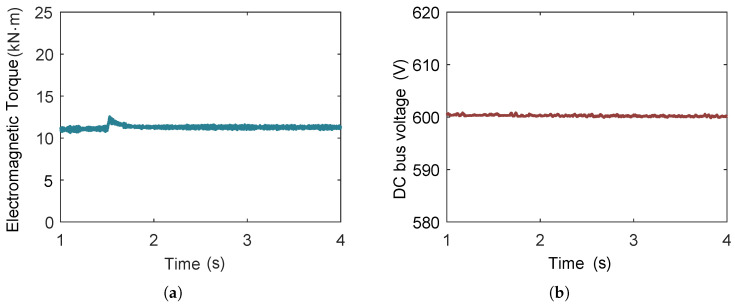
The variations in electromagnetic torque and DC bus voltage in the ORC system, when the expander rotational speed changes from 3000 rpm to 2800 rpm. (**a**) The variations in electromagnetic torque when the expander rotational speed changes from 3000 rpm to 2800 rpm. (**b**) The variations in DC bus voltage when the expander rotational speed changes from 3000 rpm to 2800 rpm.

Figure 23 illustrates the variations in the electromagnetic torque and the DC bus voltage when the rotational speed changes from 3000 rpm to 2800 rpm. As can be observed in the figure, the mechanical torque increases due to the decrease in rotational speed. Accordingly, the electromagnetic torque also increases. During the stepwise change in rotational speed of expander, the DC bus voltage can remain at the set value.

When analyzing the dynamic characteristics of electrical devices in an ORC power generation system, they are generally not simulated together with the heat exchangers. Similarly, when examining the dynamic response of the ORC system itself, the influence of electrical devices is typically neglected. Nevertheless, in certain control strategies, adjusting the expander speed becomes essential to enable the ORC system to achieve maximum output power. This adjustment is realized by regulating the rectifier current. To address the computational burden associated with such coupled simulations, the multi-time-scale framework could decompose simulation into multiple time-scales. By capturing both the fast and slow processes within appropriate time-scales, the proposed framework not only ensures accurate representation of the coupled behaviors but also substantially reduces computational effort and simulation time.

## 4. Conclusions

This paper proposes a thermal–mechanical–electrical–magnetic coupling-based multi-time-scale modeling method for ORC power generation systems. The method decomposes the system into multiple time-scales, enabling separate solution for each scale to improve computational efficiency. Applied to the dynamic modeling of a basic ORC system, the proposed approach yields simulation results with small errors. The method is further extended to model a series-type dual-loop ORC system and a series heat exchanger dual-heat-source ORC system, followed by dynamic characteristic analyses. The main conclusions are as follows:

1. The proposed multi-time-scale modeling method achieves high accuracy and significantly reduced computational demand for thermal–mechanical–electrical–magnetic coupled ORC systems. For the basic ORC model, the mean absolute error is 2.12% and the peak error is 9.53%.
2. In the series-type dual-loop ORC system, an increase in heat-source temperature has a smaller effect on the second loop, whereas a decrease in cold source temperature has a smaller effect on the first loop.
3. The dual-loop ORC system exhibits stronger anti-disturbance capability than a single-loop configuration when subjected to fluctuations in sink or heat-source conditions.
4. In the dual-heat-source ORC configuration, the overall system state and transient behavior are predominantly governed by the second (higher-quality) heat source.

The proposed framework provides a robust foundation for several practical applications. In marine engineering, it can be directly employed for the design, optimization, and control of shipboard waste–heat recovery units, assisting in the realization of low-carbon and energy-efficient vessels. Its modular structure enables integration with intelligent control systems for real-time performance prediction, disturbance compensation, and fault detection. Beyond marine use, the same methodology can be extended to land-based industrial waste-heat recovery, hybrid renewable microgrids, and energy management systems requiring fast and accurate multiphysics simulation. Future research will focus on combining the present framework with digital-twin technology and machine learning-based adaptive control to achieve online parameter identification and predictive maintenance.

## Figures and Tables

**Table 1:** The parameters of heat sources.

Parameter	Unit	Value
Exhaust gas temperature	K	535.75
Scavenge air temperature	K	422.37
Exhaust gas mass flow	kg/s	10.33
Scavenge air mass flow	kg/s	10.45

**Table 2:** The parameters of heat exchangers in series dual-loop ORC system.

Component	Parameter	Value	Unit
Evaporator (shell-and-tube)	Outer tube diameter	32	mm
	Wall thickness	2	mm
	Tube length	8.8	m
	Number of tubes	206	~
Heat exchanger (plate)	Plate length	1130	mm
	Plate width	469	mm
	Number of plates	215	~
	Plate spacing	4	mm
	Plate thickness	2	mm
Condenser (shell-and-tube)	Outer tube diameter	25	mm
	Wall thickness	2	mm
	Tube length	7.5	m
	Number of tubes	198	~

**Table 3:** The parameters of heat exchangers in multi-heat-source ORC system.

Component	Parameter	Value	Unit
Heat exchanger A (shell-and-tube)	Outer tube diameter	25	mm
	Wall thickness	2.5	mm
	Tube length	8.9	m
	Number of tubes,	817	~
Heat exchanger B (shell-and-tube)	Outer tube diameter	25	mm
	Wall thickness	2.5	mm
	Tube length	7.0	m
	Number of tubes	155	~
Condenser (shell-and-tube)	Outer tube diameter	25	mm
	Wall thickness	2.5	mm
	Tube length	7.9	m
	Number of tubes	413	~

**Table 4:** The parameters of components in multi-heat-source ORC system.

Component	Parameter	Value	Unit
Expander	Rated speed	3000	rpm
	Suction volume	0.016	$m^{3}$
	Isentropic efficiency	0.7	~
Pump	Rated speed	1500	rpm
	Rated flow rate	2.48	$m^{3}/h$
PMSG	Stator resistance	0.0086	Ω
	dq-axis inductance	6	mH
	Moment of inertia	0.029	$J/(\mathrm{kg}\cdot m^{2})$

## Data Availability

The raw data supporting the findings of this article will be made available by the authors on request.

## Abbreviations

The following abbreviations are used in this manuscript:

ORC	Organic Rankine Cycle
MPC	Model Predictive Control
T-s	Temperature–Entropy
MBM	Moving Boundary Model
PMSG	Permanent Magnet Synchronous Generator
MSC	Machine-Side Converter
GSC	Grid-Side Converter
SA	Scavenge air
EX	Exhaust gas
Symbols	
*A*	Heat transfer area
ρ	Density
$\dot{m}$	Mass flow rate
$\dot{G}$	mass flux
*h*	Enthalpy
*P*	Pressure
*N*	Rotational speed
*D*	Diameter
α	Heat transfer coefficient
*T*	Temperature
*L*	Length of the region
μ	Viscosity
$c_{p}$	Specific heat
Bo	Boling number
*k*	Thermal conductivitym
Pr	Prandtl number
FF	Filling factor
*V*	displacement volume
*v*	Specific volume
*W*	Power output
ω	Angular velocity
η	Efficiency
*R*	Resistance
*u*	Voltage
*i*	Current
ψ	Flux linkage
*J*	Moment of inertia
*S*	Switch
*C*	Capacitor
*d*	d-axis
*q*	q-axis
Subscripts	
w	wall of heat exchanger
r	working fluid
in	inlet
l	saturated liquid state of the working fluid
i	inner
g	saturated vapor state of the working fluid
out	outlet
pl	plate heat exchanger
s	single-phase zone
tp	two-phase zone
ts	tube-and-shell heat exchanger
exp	Expander
pu	Pump
ev	Evaporator
con	Condensor
e	Electric
m	Mechanical
ga	Phase A of the power grid
gb	Phase B of the power grid
gc	Phase C of the power grid

## Appendix A

### Appendix A.1

The moving boundary model of heat exchanger is presented here. For the subcooled region, the energy conservation equation is derived as follows:

(A1) $$\begin{array}{cc} \frac{1}{2}A\left({\bar{\rho }}_{1}\left(h_{\mathrm{in}}+h_{l}\right)-2\rho_{l}h_{l}\right)\frac{dL_{1}}{dt} & +\frac{1}{2}AL_{1}\left({\bar{\rho }}_{1}+\frac{1}{2}\left(h_{\mathrm{in}}+h_{l}\right)\frac{\partial {\bar{\rho }}_{1}}{\partial h}{|}_{p}\right)\frac{dh_{\mathrm{in}}}{dt}\\ & +\frac{1}{2}AL_{1}{\left\{{\bar{\rho }}_{1}\frac{dh_{l}}{dp}+\left(h_{\mathrm{in}}+h_{l}\right){\left(\frac{\partial {\bar{\rho }}_{1}}{\partial p}\right|}_{h}+\frac{1}{2}\frac{\partial {\bar{\rho }}_{1}}{\partial h}\right|}_{p}\frac{dh_{l}}{dt}-2)\}\frac{dp}{dt}\\ & ={\dot{m}}_{\mathrm{in}}h_{\mathrm{in}}-{\dot{m}}_{1}h_{l}+\pi D_{i}L_{1}\alpha_{i1}\left({\bar{T}}_{w1}-{\bar{T}}_{r1}\right) \end{array}$$

where *L* is length of the region, subscript in is the inlet, subscript l denotes the saturated liquid state of the working fluid, and subscript i represents the inner.

The pressure drop in the region is given by

(A2) $$\Delta p_{1}=f_{1}\frac{L_{1}}{d}\frac{G^{2}}{2{\bar{\rho }}_{1}}$$

The mass balance of the working fluid:

(A3) $$A\left\{\left({\bar{\rho }}_{1}-\rho_{l}\right)\frac{dL_{1}}{dt}+L_{1}\left(\frac{\partial {\bar{\rho }}_{1}}{\partial p}{{|}_{h}+\frac{1}{2}\frac{\partial {\bar{\rho }}_{1}}{\partial h}|}_{p}\frac{dh_{l}}{dp}\right)\frac{dp}{dt}+\frac{1}{2}L_{1}\frac{\partial {\bar{\rho }}_{1}}{\partial h}{|}_{p}\frac{dh_{m}}{dt}\right\}={\dot{m}}_{\mathrm{in}}-{\dot{m}}_{1}$$

The energy balance for the heat exchanger wall is formulated as follows:

(A4) $$A_{w}\rho_{w}c_{p,w}L_{1}\frac{d{\bar{T}}_{w1}}{dt}=\pi L_{1}\left\{D_{o}\alpha_{o1}\left({\bar{T}}_{\mathrm{hs}3}-T_{w1}\right)-D_{i}\alpha_{i1}\left({\bar{T}}_{w1}-{\bar{T}}_{r1}\right)\right\}$$

In the two-phase region of the heat exchanger, where the organic working fluid is in thermodynamic equilibrium between the saturated liquid and vapor states, the energy conservation equation governing the two-phase mixture can be expressed as

(A5) $$\begin{array}{cc} A\left(\rho_{l}h_{l}-\rho_{g}h_{g}\right)\frac{dL_{2}}{dt} & +A\left(1-\bar{\gamma }\right)\left(\rho_{l}h_{l}-\rho_{g}h_{g}\right)\frac{dL_{1}}{dt}\\ & +AL_{2}\left\{\bar{\gamma }\frac{d(\rho_{g}h_{g})}{dp}+\left(1-\bar{\gamma }\right)\frac{d(\rho_{l}h_{l})}{dp}-1\right\}\frac{dp}{dt}\\ & ={\dot{m}}_{1}h_{l}-{\dot{m}}_{2}h_{g}+\pi D_{i}L_{2}\alpha_{i2}\left({\bar{T}}_{w2}-{\bar{T}}_{r2}\right) \end{array}$$

where subscript g is gas, subscript $\bar{\gamma }$ is void fraction, and can be calculated by

(A6) $$\bar{\gamma }=\frac{1-\mu^{2/3}\left(1-\ln\left(\mu^{2/3}\right)\right)}{\left(1-\mu^{2/3}\right)}$$

(A7) $$\mu^{-1/3}={\left(\frac{\rho_{l}}{\rho_{g}}\right)}^{1/3}$$

The pressure drop in the region is given by

(A8) $$\Delta p_{2}=\frac{2f_{2}{G_{2}}^{2}L_{2}}{d{\bar{\rho }}_{2}}\frac{1}{\gamma_{\mathrm{tp},\mathrm{out}}-\gamma_{\mathrm{tp},\mathrm{in}}}\int_{\gamma_{\mathrm{tp},\mathrm{in}}}^{\gamma_{\mathrm{tp},\mathrm{out}}}\varphi^{2}dL$$

The mass conservation equation for the two-phase region of the heat exchanger is expressed as

(A9) $$A\left\{\left(\rho_{l}-\rho_{g}\right)\frac{dL_{1}}{dt}+\left(1-\bar{\gamma }\right)\left(\rho_{l}-\rho_{g}\right)\frac{dL_{2}}{dt}+L_{2}\left(\bar{\gamma }\frac{d\rho_{g}}{dp}+\left(1-\bar{\gamma }\right)\frac{d\rho_{l}}{dp}\right)\frac{dp}{dt}\right\}={\dot{m}}_{1}-{\dot{m}}_{2}$$

The energy balance for the heat exchanger wall in the two-phase region is governed by the following expression:

(A10) $$A_{w}\rho_{w}c_{p,w}L_{2}\frac{d{\bar{T}}_{w2}}{dt}=\pi L_{2}\left\{D_{o}\alpha_{o2}\left({\bar{T}}_{\mathrm{hs}2}-T_{w2}\right)-D_{i}\alpha_{i2}\left({\bar{T}}_{w2}-{\bar{T}}_{r2}\right)\right\}$$

The energy conservation equation for the superheated vapor region of the heat exchanger is formulated as follows:

(A11) $$\begin{array}{cc} \; & A\left(\rho_{g}h_{g}-\frac{1}{2}{\bar{\rho }}_{3}\left(h_{g}+h_{\mathrm{out}}\right)\right)\left(\frac{dL_{1}}{dt}+\frac{dL_{2}}{dt}\right)\\ \; & +\;AL_{3}\left\{\frac{1}{2}\left(h_{g}+h_{\mathrm{out}}\right)\left(\frac{1}{2}\frac{\partial {\bar{\rho }}_{3}}{\partial h}{{|}_{p}\frac{dh_{g}}{dp}+\frac{\partial {\bar{\rho }}_{3}}{\partial p}|}_{h}\right)+\frac{1}{2}{\bar{\rho }}_{3}\frac{dh_{g}}{dp}-1\right\}\frac{dp}{dt}\\ \; & +\;A\left(\frac{1}{2}{\bar{\rho }}_{3}L_{3}+\frac{1}{4}\frac{\partial {\bar{\rho }}_{3}}{\partial h}{|}_{p}\left(h_{g}+h_{\mathrm{out}}\right)L_{3}\right)\frac{dh_{\mathrm{out}}}{dt}\\ \; & =\;{\dot{m}}_{2}h_{g}-{\dot{m}}_{\mathrm{out}}h_{\mathrm{out}}+\pi D_{i}L_{3}\alpha_{i3}\left({\bar{T}}_{w3}-{\bar{T}}_{r3}\right) \end{array}$$

The mass continuity equation for the superheated vapor region of the heat exchanger is governed by the following expression:

(A12) $$\begin{array}{cc} A\left(\left(\rho_{g}-{\bar{\rho }}_{3}\right)\frac{dL_{1}}{dt}+\left(\rho_{g}-{\bar{\rho }}_{3}\right)\frac{dL_{2}}{dt}\right)+AL_{3}\left(\frac{1}{2}\frac{\partial {\bar{\rho }}_{3}}{\partial h}{|}_{p}\frac{dh_{g}}{dp}+\frac{\partial {\bar{\rho }}_{3}}{\partial p}{|}_{h}\right)\frac{dp}{dt}+\frac{1}{2}AL_{3}{\left.\frac{\partial {\bar{\rho }}_{3}}{\partial h}\right|}_{p}\frac{dh_{\mathrm{out}}}{dt} & ={\dot{m}}_{2}-{\dot{m}}_{\mathrm{out}} \end{array}$$

The energy balance for the heat exchanger wall in the superheated region:

(A13) $$A_{w}\rho_{w}c_{p,w}L_{3}\frac{d{\bar{T}}_{w3}}{dt}=\pi L_{3}\left\{D_{o}\alpha_{o3}\left({\bar{T}}_{\mathrm{hs}1}-T_{w3}\right)-D_{i}\alpha_{i3}\left({\bar{T}}_{w3}-{\bar{T}}_{r3}\right)\right\}$$

### Appendix A.2

The heat transfer coefficients for the superheated and subcooled regions in plate heat exchangers are calculated as follows [35]:

(A14) $$\alpha_{s,\mathrm{pl}}=0.023\frac{k_{r}}{D_{\mathrm{pl}}}Re_{r}^{0.8}Pr_{r}^{0.4}{\left(\frac{\mu_{r}}{\mu_{w,r}}\right)}^{0.14}$$

where subscript s is the single-phase zone. The heat transfer coefficients of two-phase zone is deduced by [36]:

(A15) $$\alpha_{\mathrm{tp},\mathrm{pl}}=1.926\frac{k_{r}}{D_{\mathrm{pl}}}Bo_{\mathrm{eq}}^{-0.3}Re_{\mathrm{eq}}^{0.5}Pr_{\mathrm{eq}}^{1/3}\left[\left(1-x\right)+x{\left(\frac{\rho_{l}}{\rho_{g}}\right)}^{0.5}\right]$$

where subscript s is the two-phase zone.

The heat transfer coefficients for the superheated and subcooled regions in tube and shell heat exchanger could be calculated:

(A16) $$\alpha_{s,\mathrm{ts}}=0.023\frac{k_{r}}{D_{\mathrm{st}}}Re_{r}^{0.8}Pr_{r}^{a}$$

where subscript ts is tube-and-shell heat exchanger.

The heat transfer coefficients of two-phase zone in tube and shell heat exchanger is deduced by

(A17) $$\alpha_{\mathrm{tp},\mathrm{ts}}=0.023{\left[\dot{G}\left(1-x\right)\frac{d}{\mu_{l}}\right]}^{0.8}\frac{Pr^{0.4}\lambda_{l}}{d}E$$

The condensation heat transfer coefficients in the plate heat exchanger can be expressed by

(A18) $$\alpha_{\mathrm{con},\mathrm{pl}}=4.118\frac{k_{r,l}}{D_{\mathrm{pl}}}Re_{\mathrm{eq}}^{0.4}Pr_{l}^{1/3}$$
